# Design and optimization of crocetin loaded PLGA nanoparticles against diabetic nephropathy via suppression of inflammatory biomarkers: a formulation approach to preclinical study

**DOI:** 10.1080/10717544.2019.1642417

**Published:** 2019-09-14

**Authors:** Xiaodong Yang

**Affiliations:** 1 Department of General Medicine, Zhumadian Central Hospital, Zhumadian, China

**Keywords:** Crocetin, PLGA loaded nanoparticles, antidiabetic, protein kinase C, antifibrotic, streptozotocin, NF-kB

## Abstract

Diabetic nephropathy (DN) is a serious complication of diabetes mellitus whose expand process is linked with the fibrosis, renal hypertrophy and inflammation. The current study was to formulate and optimize the nano-formulation of crocetin (CT-PLGA-NPs) against Streptozotocin-induced renal nephropathy in rats. Double emulsion evaporation technique was used for the preparation of CT-PLGA-NPs. CT-PLGA-NPs were scrutinized for polydispersity index, size, gastric stability, entrapment, drug-loading capacity and *in-vitro* drug release and *in vivo* preclinical study. Single intraperitoneal injection of streptozotocin (STZ) (55 mg/kg) and rats were divided into different group. Renal function and metabolic parameters of urine and serum were estimated. Fibrotic protein, renal pro-inflammatory cytokines and degree of renal damage expression were also determined. We also estimated the fibronectin, type IV collagen and transforming growth factor-β1 for a possible mechanism of action. Crocetin supplement (10 mg/kg) and CT-PLGA-NPs exhibited the accumulation of the drug in kidney and liver of diabetic rats. Crocetin reduced the BGL and enhanced plasma insulin and body weight. Dose dependent treatment of crocetin significantly (*p* < .001) down-regulated the expression of renal tumor necrosis factor-α (TNF-α), interleukin-6 (IL-6), interleukin (IL)-1β (IL-1β) and Monocyte Chemoattractant Protein-1 (MCP-1). Crocetin significantly (*p* < .001) altered the expression of fibronectin, type IV collagen, and transforming growth factor-β1 (TGF-1β). Crocetin significantly (*p* < .001) down-regulated the protein kinase C activity and the expression of nuclear factor κB (NF-κB) p65 activity and protein production in renal tissue. On the basis of the available result, we can conclude that nano-formulation of crocetin could attenuate the diabetic nephropathy via antifibrotic and anti-inflammatory effect.

## 1. Introduction

As a resultant of lifestyle changes and socioeconomic expansion, the incidence of diabetes has been globally raised in the last few decades. At present times, diabetes mellitus (DM) has become a global health problem in China and treatment for DM and its complications is urgently needed (Martyn-Nemeth et al., 2016). Diabetic nephropathy (DN) is the commonly occurring complication of DM and major health problem worldwide (Cordonnier et al., 2001). Fibrosis and inflammation, both play an important role in the expansion of DN, which depreciates the severity of renal functions and mortality of diabetes (Bilous, 2001). DN is the end stage of renal disease and leading to death in diabetic patients is categorized pathologically via stimulation the accumulation of extracellular matrix (ECM) and hypertrophy (Kolset et al., 2012). The accumulation of ECM in DN results in irreversible deterioration of renal function, tubulointerstitial fibrosis, and expansion of mesangial (Mason and Wahab, 2003). Previous studies suggest that during the diabetic conditions, firstly start the accumulation of ECM and attributable to hemodynamic changes, advanced glycation end products and transforming growth factor β1 (TGF- β1), the important cellular and molecular mechanism responsible for this are yet to be solved (Chang et al., 2016). Hyperglycemia parse Hyperlipidaemia, hypertension, metabolic syndrome manifestation of obesity and hyperglycemia are strongly linked with chronic renal disease (Chang et al., 2016). Glomerulosclerosis and tubulointerstitial fibrosis, both are involved in the pathogenesis of nephropathy in type 2 diabetes, induced by the inflammation, oxidative stress, secretion of profibrotic factors (transforming growth factor β1) and epithelial-mesenchymal transition (Chang et al., 2016). Clinical studies of diabetic nephropathy patients showed the over-production of pro-inflammatory cytokines and chemokines viz., tumor necrosis factor (TNF-α), interleukin (IL-1β) and monocyte chemoattractant protein-I (MCP-1) (Chang et al., 2016). These inflammatory factors not only boosted the diabetic linked renal inflammation but also disturb the systemic immune functions. The boosting level of TGF-β1 leads to the massive production of the extracellular matrix such as type IV collagen and fibronectin (Amann et al., 2003). The activation of NF-κB and increased protein kinase C (PKC) activity boosted the fibrotic and inflammatory progression under diabetic conditions. Subsequently, renal replacement or renal dialysis is essential for a patient with diabetic nephropathy to endure (Amann et al., 2003). Several studies suggest that the inflammatory process play an important role in the pathogenesis of DN. Renal tubulointerstitium and infiltration of inflammatory cells in the glomeruli played an important role in the experimental rodent and human diabetic patient. Inflammatory mediators such as MCP-1, which arbitrates the infiltration of monocytes/macrophages, have been involved in the pathophysiology of DN (Muñoz-Félix et al., 2013). Based on the previous studies, alteration of inflammatory reaction is targeted to be developed progression and expansion of DN, and some anti-inflammatory and immunosuppressive drugs have shown a protective effect in DN (Chang et al., 2016). Yet, chronic use of the above discussed drugs in the clinical field is not sufficient due to numerous side effects. While much attention is needed for controlling the blood glucose level during diabetes and prevent the progression of kidney disease. Conversely, recent clinical and experimental studies have exhibited that hyperglycemia induces inflammation, lipid accumulation and oxidative stress, which start the dysfunction of renal tissue via triggering the multiple signaling pathways and these may be critical factors in the DN pathogenesis. Crocetin solutions have limitations such as poor oral bioavailability and instability to pH variations. Several researchers have developed various approaches to enhances the bioavailability of drug molecules by nano-sizing of drugs, and loaded into lipid vesicles such as nanoemulsion, nano-lipidic carrier, etc. In the design of oral delivery of drugs, polymeric nanoparticles have attracted increased attention. A variety of synthetic or natural polymers have been employed to fabricate polymeric nanoparticles (Bhatt et al., 2017).

Previous studies suggest that natural carotenoids are highly pigmented phytoconstituents that consist of isoprene unit (8) and commonly found as oxygenated or hydrocarbons phytoconstituent. Studies suggest that carotenoids possess immunomodulating, anti-mutagenic and anti-carcinogenic effects. Carotenoids and retinoids have been shown to reduce the growth of certain type of cancer cells such as neuroblastoma, leukemia, colon cancer and breast cancer. However, carotenoids and retinoids and their synthetic derivatives were proposed for experimental chemoprevention and also used for the treatment of cancer. But, to date as my knowledge, no relationship found between the ingestion of carotenoid-containing fruits and diabetes risk.

There are many phytoconstituents from natural products that showed the tumor-suppressing activity, thus being potentially useful in the treatment of diabetes. Various extracts showed the cytotoxic effect in the cancer cells and cancer animal model. Saffron, *Crocus sativus* L., was used to treat various diseases especially cancer and diabetes by Chinese, Indian and Arabian people in the ancient times. Saffron contains the carotenoids in addition to riboflavin. Crocetin is the important phyto-constituent of saffron and showed significant potential as an anti-tumor agent in rodent modes and cell culture systems.

PLGA is very commonly used in the development of various types of nanoparticles for the drug delivery system which is approved from food and drug administration (FDA) for human use. PLGA nanoparticles provide better stability, the solubility of the drug with greater therapeutic effects. Various researchers suggest that PLGA is a powerful biocompatible polymer due to its biodegradable nature, and its hydrolysis produces glycolic acid and lactic acid. These two monomers are endogenous and further metabolized via the body through the Krebs cycle and removed in the form of water and carbon dioxide, which are responsible for the insignificant toxic effect. Various published literature available, which showed that PLGA nanoparticles for encapsulations of various anti-diabetic drug and successfully delivery as *in vitro* and *in vivo* (Savioli Lopes et al., 2012). Furthermore, the literature suggests that the polymeric nanoparticles increased the drug target efficiency and bioavailability (Pirooznia et al., 2012). This paper mainly focused to summarize the importance of PLGA nanoparticles, a new vehicle for crocetin by highlighting the enhanced properties in terms of (i) levels of inflammatory cytokines of renal tissue, ii) on antioxidant enzymes, iii) on renal fibrotic factor iv) on NF-κB p50, NF-κB p65, and PKC activity.

## 2. Material and methods

### 2.1. Chemical

Crocetin (99%) was purchased from the Sigma Aldrich Co. (St. Louis, MO, USA). Other chemicals used in the experimental study were of highest purity and procured from reputed vendor.

### 2.2. *In vitro* α-glucosidase activity

Kumar et al. reported method was used for the estimation of the inhibitory effect of crocetin on α-glucosidase with minor modification (Kumar et al., 2016). Briefly, various concentration of crocetin was mixed with the (20 μl solution) enzymes, which contain the α-glucosidase (0.8 U/ml) in phosphate buffer saline (0.01 M) and incubated at 37 °C at room temperature for 20 min and for the termination the reaction Na_2_CO_5_ (0.2 M) was added and again incubated at 37 °C for 15 min and absorbance was recorded at 430 nm using the 96 well microtiter plate. The final result was obtained as % inhibition of α-glucosidase using the current formula:

$$\text{Inhibition }\left(\%\right)={\text{ Control}}_{\text{Absorbance}}-{\text{Test}}_{\text{Abosrbance}}\text{ × }100/{\text{Control}}_{\text{Absorbance}}$$

### 2.3. α-amylase activity

Ahmed et al., the method was used with minor modification for the determination of α-amylase activity (Ahmed et al., 2014). Briefly, samples such as crocetin and acarbose were mixed incubated with 20 mM sodium phosphate (pH = 6.7) for 5 min and starch was used to makeup the volume up to 2 ml and the sample was again re-incubated at room temperature for 5 min and 1 ml of di-nitro salicylic acid was added. The reaction mixture was kept in boiling water bath for 5 min. After that, ice was used for cooling the reaction and deionized water was added and the absorbance of reaction mixture was measured at 540 nm. The inhibition of percentage was scrutinized using the above formula.

### 2.4. Formulation of crocetin loaded PLGA nanoparticles

Briefly, double emulsion solvent method with minor modification was used for the preparation of polymeric nanoparticles (Bhatt et al., 2017; Kumar et al., 2017). For the external and internal aqueous phase, Span 60 and Tween 80 was used and for the external aqueous phase, polyvinyl alcohol (1%) used as a stabilizer. For W_1_/O emulsion, crocetin was soluble in dichloromethane (10–20% w/w) solution of Span 60, PLGA and Tween 80. After that, the solution was sonicated using the ultrasonicator for 20 sec at 50 W on ice cold water bath (to reduce the temperature). Additionally, the prepared emulsion was gradually deionized by adding the polyvinyl alcohol at various concentrations. Again, the prepared emulsion was sonicated for 20 sec to prepare the second emulsionW_1_/O/W_2_. In the continuation, the emulsion was again stirred at 1500 rpm under the optimum temp for complete removal of dichloromethane. The prepared nanoparticles of crocetin were harvested and washed and re-dispersed in the deionized water at 18000 rpm for 20 min and the prepared nanoparticles were again washed to remove the surfactant and drug. The prepared nanoparticles were lyophilized for 1 day to obtain the powder and stored at −20 °C for further use.

### 2.5. Characterization of CT-PLGA-NPs

#### 2.5.1. Particle size (PS), polydispersity index (PDI) and zeta (ζ) potential

For the estimation of particle size (PS) and polydispersity index (PDI), particle size analyzer was used with dynamic light scattering (DLS) method. The nano-formulation was 200 times diluted with aqueous phase followed by vigorous shaking to obtain 100–300 kilo counts per second. DLS was used for the estimation of ζ potential using the Zetasizer ZS 90 (M/s Malvern Instruments, Worcestershire, UK).

#### 2.5.2. Scanning electron microscopy (SEM)

SEM spectroscopy was used for the estimation of the surface morphology and microscopic evaluation of prepared CT-PLGA-NPs.

#### 2.5.3. Drug loading capacity and encapsulation efficiency

Drug loading capacity was used for the estimation of the drug content in the present nanoparticles after the separation from the medium. The entrapment efficiency is used for the estimation of entrapped/adsorbed into CT-PLGA-NPs. The entrapment efficiency (EE) and loading capacity of CT-PLGA-NPs were estimated by separating free crocetin from the PLGA-NPs at 12,000 rpm for 20 min. The entrapment efficiency and drug loading capacity was estimated using the following formula:

(1) $$\text{Drug loading capacity }=\;\frac{\mathrm{Entrapped Drug}}{\mathrm{Nanoparticles weight}}\times 100$$

(2) $$\mathrm{EE}\;(\%)\;=\frac{\begin{array}{l} \mathrm{Total}\;\mathrm{amount}\;\mathrm{of}\;\mathrm{crocetin}\\ -\mathrm{Amount}\;\mathrm{of}\;\mathrm{crocetin}\;\mathrm{into}\;\mathrm{talsupernatant} \end{array}}{\mathrm{Amount}\;\mathrm{of}\;\mathrm{rutin}}\times 100$$

#### 2.5.4. *In vitro* gastrointestinal stability

For the stability of CT-PLGA-NPs in the gastrointestinal tract, the *in-vitro* technique was used. The optimized CT-PLGA-NPs were subjected to 200 ml of simulated gastric fluid for 3 h and simulated intestinal fluid for 9 h. At the time interval of 3 h, the sample (1 ml) was kept in the cuvette for the estimation of entrapment efficiency, particle size, and zeta potential.

#### 2.5.5. *In-vitro* drug release study

The *in-vitro* drug release was used for the estimation of the release pattern of CT-PLGA-NPs via using the dialysis method. Briefly, CT-PLGA-NPs were incubated in phosphate buffer saline (50 ml, pH = 7.4) at 37 ± 2 °C. The aliquot samples were collected at different time intervals and the quantity of crocetin was measured to estimate the cumulative drug release vs. time.

### 2.6. Animal

Swiss albino Wistar rats (4–5 weeks old, 125–150 g) were obtained from the Departmental animal house and stored in the standard environmental condition. The rats were housed in the standard laboratory condition 22 ± 5 °C, 12 h light/dark cycle; water and standard rat pellet were supplied *ad libitum*. The current experimental protocol was reviewed and endorsed by the Institutional Animal Care Committee.

### 2.7. Induction of diabetes

Single intraperitoneal injection (50 mg/kg, b.w.) of Streptozotocin (STZ) was used for the induction of diabetes (Tsai et al., 2012). Briefly, STZ was dissolved in the citrate buffer (pH = 4.5) and injected into overnight fasted rats. One touch blood glucose meter was used for checking the blood glucose level (BGL) and rats having BGL ≥250 mg/dL were used for the current experimental study (Kumar et al., 2013).

### 2.8. Experimental protocol

After successful induction of diabetes, the rats were randomly divided into five groups and each group contained 10 rats. The rats were divided into the following groups such as Gp I: normal control, Gp II: diabetes control Gp III–V received crocetin (2.5, 5 and 10 mg/kg, b.w.). All group rats had free access to water and food at all times. Feed volumes, body weight, and consumed water were recorded at regular interval. BLG and plasma insulin level were estimated at week 0 (start of the experimental study) and week 16 (end of the experimental study) (Vaishya et al., 2008). After the completion of the experimental study, the rats were killed with an excess of anesthesia, and blood samples were collected in all group rats and plasma was separated from the erythrocytes quickly. Rodent tissue such as kidney and liver were quickly removed and weighted and each tissue was homogenized using the ice-cold phosphate buffer saline (2 mL) at pH7.2 and passed through the Whatman filter paper and the filtrate was separately collected.

### 2.9. Biochemical parameters

Biochemical parameters such as blood glucose level, plasma insulin, albumin, total protein, blood urea nitrogen (BUN) and creatinine clearance were estimated via using the available kit via using the manufacturer's instruction (Span Diagnostic, India).

### 2.10. Estimation of crocetin content

High-performance liquid chromatography (HPLC) techniques were used for the estimation of crocetin content in different organs (renal, hepatic tissue and plasma samples) in the rodent. Briefly, the different samples were incubated with a mixture containing 0.78 mM sodium acetate buffer (pH 4.8), 0.1 Mm ascorbic acid and 89.4 units/mLglucuronidase. For partition of the sample, ethyl acetate was used, followed by vortexing for 60 s and centrifuged at 6000 *g* for 15 min. After successfully removing the ethyl acetate layer, the remaining residue was re-constituted with methanol and forwarded to the HPLC analysis (Castro-Perez, 2007).

### 2.11. Estimation of pro-inflammatory cytokines

Perfused hepatic and renal tissue were homogenized in Tris-HCl buffered solution (10 mM) containing ethylenediaminetetraacetic acid (1 mM), NaCl (2 M), Tween 80 (1%) and phenylmethanesulfonyl fluoride (1 mM) and centrifuged at 10000 *g* for 20 min at 4 °C. The supernatant was further used for the estimation of pro-inflammatory cytokines such as MCP-1, TNF- α, IL-6 and IL-1 β via using the instruction provided by the ELISA kit manufacturers (RayBiotech Life, Norcross, GA, USA).

### 2.12. NF-κB p50/65 assay

Commercially available kits were used for the estimation of NF-κB p50/65 DNA binding activity (Chemicon International Co., Temecula, CA, USA). Primary polyclonal anti- NF-κBp50/p65 antibody was used for the scrutinized the binding activated NF-κB, a secondary antibody conjugated with horseradish peroxidase, and the 3,3′,5,5′-tetramethylbenzidine substrate, and the absorbance was estimated at 450 nm and the values were presented as milligram of protein.

### 2.13. Estimation of fibronectin, TGF-β1, type IV collagen and urinary albumin

ELISA kits were used for the estimation of Fibronectin, TGF- β1, Type IV Collagen and urinary Albumin level in the renal tissue. Briefly, the renal cortex was homogenized in ice-cold phosphate buffer saline containing Tween 20 (0.05%) and centrifuged at 9000 g rpm for 20 min at 4 °C and supernatants were further used for the estimation of Fibronectin, TGF-β1 and Type IV collagen concentration via using the ELISA kits (Sigma Aldrich, USA).

### 2.14. Estimation of kidney glomeruli PKC activity

In brief, the renal tissue sample was homogenized in the ice-cold medium of HEPES. The glomeruls was successfully isolated from the renal tissue by removing the capsules and step by step passed through the various numbers of sieves. After washing with RPMI1640 medium (containing HEPES 20 mM, pH = 7.4) and mixing with salt solution (0.4 mM potassium phosphate, 137 mM NaCl, 0.3 mM sodium phosphate, 5.4 mM KCl, 5.5 mM glucose, 25 mM β-glycerophosphate, 5.5 mM glucose, 2.5 mM CaCl_2_, 5 mM EGTA, 10 mM MgCl_2_ and HEPES 20 mM), glomerulus was again incubated with salt solution for 15 min in the presence and absence of PKC-specific substrate (100 μM) and also adding the digitonin (5 mg/mL) and ATP (1 mM) with γ-[32P]ATP (<1500 ppm/pmol) and trichloroacetic acid (5%) was used for terminate the reaction. The sample was transferred onto the P81 phosphocellulose paper and washed 4 times with phosphoric acid (1%) and one time with acetone. Scintillation counting was used for the determination of radioactivity incorporated into the substrate. Glomerular PKC activity was estimated in correspond to protein estimation.

### 2.15. Estimation of mRNA expression

Trizol reagent was used for isolate the total RNA. RNA (1 mg) was used to generate cDNA, which was augmented using Taq DNA polymerase. PCR was performed in 50 μL of reaction mixture containing Taq DNA polymerase buffer (200 mMdNTP, 2.5 mM MgCl_2_, 50 mM KCl, 20 mMTris-HCl, pH 8.4, 0.5 mM of each primer) and Taq DNA polymerase (2.5 U). The specific oligonucleotide primers are presented in Supplementary Table 1. Real-time sequence system was used for estimation of the generated fluorescence for each cycle and mRNA concentration was estimated as a percentage of DM group rats.

### 2.16. Statistical analysis

All the data presented in the current experimental study in the form of mean ± SD. *Post hoc* testing was used for the estimation of statistical significance via using the GraphPad Prism. *p* < .05 was considered statistically significant.

## 3. Result

### 3.1. *In vitro* α-glucosidase activity and α-amylase activity

Supplementary Table 3 exhibited the inhibitory effect of crocetin on the α-glucosidase activity and α-amylase enzymes. Crocetin showed the reduction of α- glucosidase, α-amylase and DPPIV (IC_50_ = 65.45 ± 2.35, 43.45 ± 1.93 and 35.43 ± 1.34 μg/mL), respectively.

### 3.2. Formulation and optimization of Crocetin-PLGA-NP

#### 3.2.1. Characterization of the optimized CT-PLGA-NPs

##### 3.2.1.1. PS & PDI

**Figure 1.**
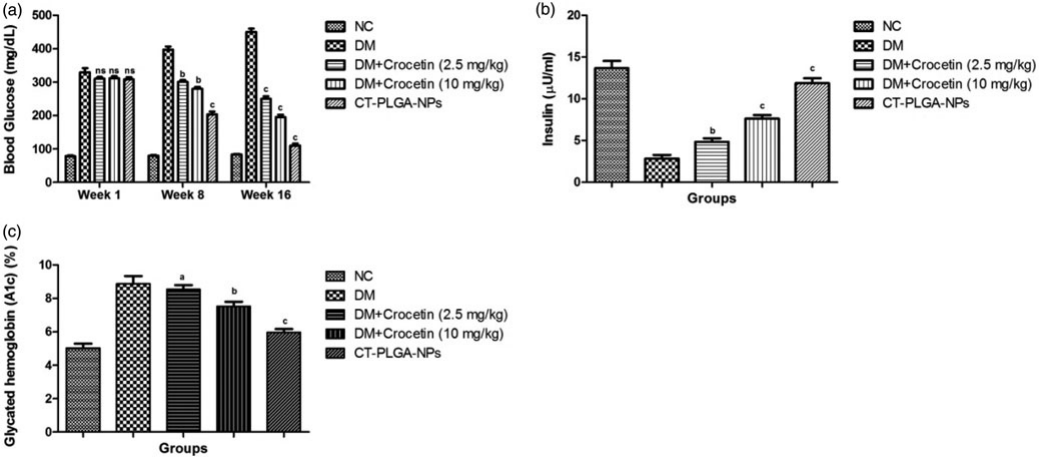
The effect of crocetin and CT-PLGA-NPs on biochemical parameters of diabetic and non-diabetic rats. (a) blood glucose level, (b) plasma insulin and (c) glycated hemoglobin method as described in material and methods. ns: non significant. All values are presented as mean ± SEM. Statistical analysis by one-way ANOVA followed by Dunnett’s multiple comparison. ^a^*p* < .05, ^b^*p* < .01 and ^c^*p* < .001.

Figure 1(b) showed the PS distribution of optimized CT-PLGA-NPs formulation with the size 218 nm and PDI 0.25. The prepared CT-PLGA-NPs had the monodisperse nature. The ζ-potential of CT-PLGA-NPs was in the range of −21.8 and −23.1 mV demonstrating the negative charge of the nano-formulation. Normally, PLGA-NPs showed negative ζ-potential, which is similarly presented in the above-discussed formulation. This is occurring due to the presence of free carboxylic group present in the PLGA polymer on the surface.

##### 3.2.1.2. TEM imaging

Supplementary Figure 1a showed the image of TEM, which suggests the spherical nano-structured nature of the particles.

### 3.3. Drug-loading efficiency & entrapment efficiency

The prepared CT-PLGA-NPs formulation showed 6.51% (±0.14) drug loading capacity and 75.39% (±1.62) entrapment efficiency, which exhibited a suitable concentration in drug and polymer. Moreover, this also demonstrated the effective method for the preparation of NPs.

### 3.4. *In vitro* gastrointestinal stability

Supplementary Table 2 showed the value of ζ-potential, entrapment efficiency and PS of the optimized CT-PLGA-NPs after being subjected to different gastrointestinal fluids.

### 3.5. *In vitro* drug-release study

Supplementary Figure 2 showed the in-vitro drug release pattern of prepared CT-PLGA-NPs. *In-vitro* drug release study showed the biphasic pattern of nono-formulation with initial burst release founded in first 4 h, followed by sustained drug release pattern with a maximum of 75% drug released after 48 h. This can be documented to the survival of CT encapsulated in PLGA NPs matrix, which follows a slow release pattern via surface erosion mechanism.

### 3.6. Effect of crocetin in organs and plasma

Supplementary Figure 3 revealed the deposition of crocetin content in different tissues and plasma. Figure 3 clearly showed that the drug deposition was significantly increased in the diabetic rats kidney and liver tissue. Rats from CT-PLGA-NPs treated group demonstrated the deposition of drug in hepatic tissue as 1.68 ± 0.003 mg/kg and 0.95 ± 0.008 mg/kg in renal tissue, respectively. A similar result was observed in the plasma, CT-PLGA-NPs treated group rats demonstrated the 0.27 ± 0.005 mg/kg found in the plasma.

### 3.7. Effect of crocetin on food intake, water intake, body weight, organ weight and urine output

Food and water intake was considered as another parameter for the treatment of diabetes and expansion of disease. During diabetes, increased excessive thrust and similar result was found in the DM control group rats. DM control group rats showed the increased water intake (18.87 ± 1.84 ml/day) (Supplementary Figure 4a) and food intake (16.74 ± 2.41 g/day) (Supplementary Figure 4b), which was almost 4 times higher as compared to normal control. Concentration-dependent treatment of crocetin significantly (*p* < .001) decreased the water and food intake at the end of the study. CT-PLGA-NPs showed the water intake (10.67 ± 1.73 ml/day) and food intake (8.92 ± 0.89 g/day) at the end of the experimental study.

Supplementary Figure 4c illustrated the body weight of different group of rats. Normal control group rats showed increased body weight with growth gain rate of 1.35 mg/kg/day. Diabetic control group rats showed the increased body weight at end of the experimental study with growth gain rate 0.44 mg/kg/day and on the other hand, crocetin dose 2.5, 5 and 10 mg/kg, b.w. exhibited the increased body weight with growth gain rate 0.58, 0.78 and 1.55 mg/kg/day, respectively.

At the end of the study, we estimated the organ weight of all group rats. DM group rats exhibited the enhancement of organ weight such as liver (16.84 ± 0.82 mg/kg), kidney (10.63 ± 0.69 mg/kg) and heart (1.89 ± 0.06), respectively. The liver and kidney tissue weight were increased and heart tissue weight was decreased in the DM group rats as compared to normal control group rats. Crocetin (10 mg/kg, b.w.) treated group rats showed significant (*p* < .001) alteration in the organ weight including liver (11.03 ± 0.67 mg/kg), kidney (8.12 ± 0.42 mg/kg) and heart (3.45 ± 0.09) at end of the experimental study (Supplementary Figure 4d).

Supplementary Figure 4e demonstrated the urine output of all group rats. DM group rats exhibited the increased urinal output (8.94 ± 1.35 ml/day) due to the diseases and dose-dependent treatment of crocetin (2.5 and 10 mg/kg, b.w.) and CT-PLGA-NPs significantly (*P* < 0.001) decreased (8.36 ± 0.98, 6.91 ± 0.78 and 5.4 ± 0.63 ml/day) the urinal output.

### 3.8. Effect of blood glucose level, insulin, and glycated hemoglobin

During diabetes mellitus, there is an increase in the blood glucose level, plasma insulin, and glycated hemoglobin. A similar result was found in our experimental study. Figure 1(a) showed the increased blood glucose level at a different time interval. Normal control group rats showed the unchanged blood glucose level at the end of the experimental study. DM group rats demonstrated the increased blood glucose level from 329 ± 30.45 mg/dL to 455.3 ± 23.12 mg/dL at the end of the experimental study (week 16). Dose-dependent treatment of crocetin (2.5 and 10 mg/kg) and CT-PLGA-NPs significantly (*p* < .001) down-regulated the blood glucose level (310.34 ± 15.76 mg/dL), (311.3 ± 14.76 mg/dL) and (309 ± 11.72 mg/dL) to (249.53 ± 12.83 mg/dL), (195.4 ± 12.34 mg/dL) and (110.3 ± 10.34 mg/dL) at a dose of 2.5, 5 and 10 mg/kg, respectively.

Another parameter i.e. plasma insulin level was decreased during the experimental study. Normal control group rats showed that the normal plasma insulin level and DM group rats showed that the reduced plasma insulin level at end of the experimental study and dose-dependent treatment of crocetin significantly (*p* < .001) up-regulated the plasma insulin level. CT-PLGA-NPs showed the increased plasma insulin (12.10 ± 1.28 µU/ml) at the end of the experimental study (Figure 1(b)).

An opposite trend was observed in the glycated hemoglobin level of DM group rats and dose-dependent treatment of crocetin significantly (*p* < .001) reduced the glycated hemoglobin (Figure 1(c)).

### 3.9. Effect of crocetin on levels of inflammatory cytokines of renal tissue

**Figure 2.**
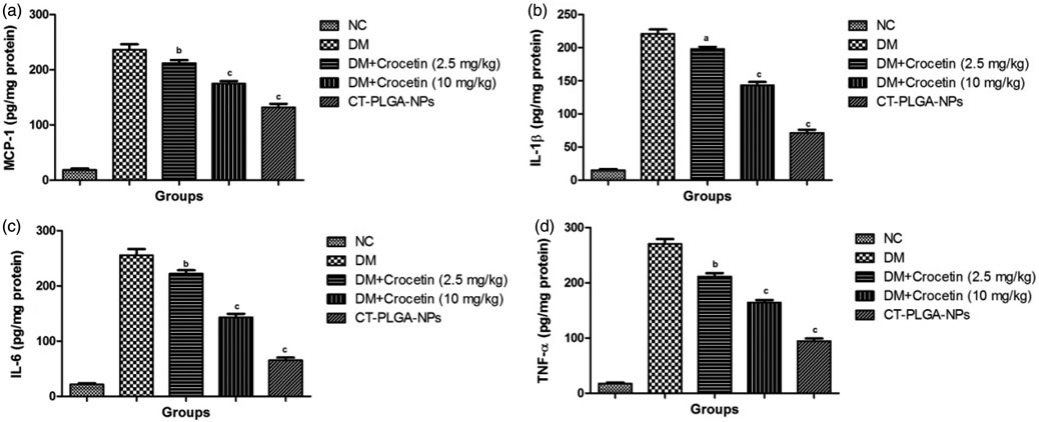
The effect of crocetin and CT-PLGA-NPs on inflammatory cytokines of diabetic and non-diabetic rats. (a) MCP-1, (b) IL-1β, (c) IL-6 and (d) TNF-α method as described in material and methods. MCP-1: Monocyte Chemoattractant Protein-1; IL-1β: Interleukin-1β; IL-6: Interleukin-6; TNF-α: Tumor necrosis factor-α; ns: non significant. All values are presented as mean ± SEM. Statistical analysis by one-way ANOVA followed by Dunnett’s multiple comparison. ^a^*p* < .05, ^b^*p* < .01 and ^c^*p* < .001.

Several researchers suggest that the level of inflammatory cytokines was considerably boosting during diabetes and a similar result was found in the current experimental study. Normal control group rats showed the normal level of inflammatory cytokines such as MCP-1 (18.29 ± 1.83), IL-1β (15 ± 1.01), IL-1β (21.83 ± 1.85) and TNF-α (17.02 ± 1.03) and DM group rats showed the increased level of MCP-1 (236.94 ± 5.83), IL-1β (221.09 ± 5.12), IL-1β (255.2 ± 4.83) and TNF-α (271 ± 3.94), respectively. On the other hand, crocetin (10 mg/kg b.w.) treatment exhibited the reduced level of MCP-1 (132 ± 3.25), IL-1β (71.92 ± 2.83), IL-6 (65 ± 2.12) and TNF-α (94.12 ± 1.85) at end of the experimental study (Figure 2(a–d)).

**Figure 3.**
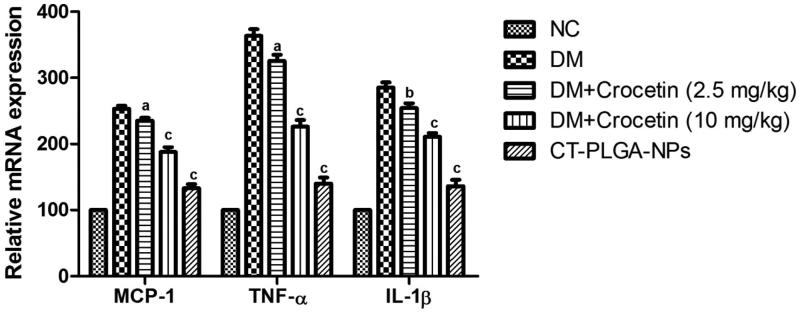
The effect of crocetin on relative expression of inflammatory cytokines in diabetic and non-diabetic rats. MCP-1: Monocyte Chemoattractant Protein-1; IL-1β: Interleukin-1β; TNF-α: Tumor necrosis factor-α; ns: non significant. All values are presented as mean ± SEM. Statistical analysis by one-way ANOVA followed by Dunnett’s multiple comparison. ^a^*p* < .05, ^b^*p* < .01 and ^c^*p* < .001.

Figure 3 demonstrated the effect of the crocetin on the mRNA expression of inflammatory cytokines. DM group rats showed the increased inflammatory cytokines mRNA expression as compared to the normal control group rats. Crocetin (2.5 and 10 mg/kg b.w.) treatment showed the lowered inflammatory cytokines mRNA expression but CT-PLGA-NPs significantly (*p* < .001) reduced the inflammatory cytokines mRNA expression as compared to DM group rats.

**Figure 4.**
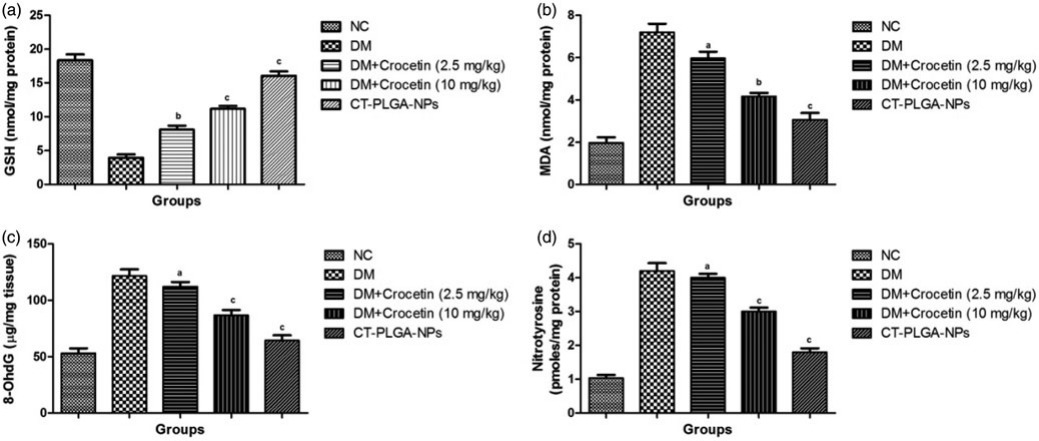
The effect of crocetin on antioxidant parameters of diabetic and non-diabetic rats. (a) GSH, (b) MDA, (c) 8-OhdG and (d) Nitrotyrosine method as described in material and methods. GSH: Glutathione; MDA: Malonaldehyde; 8-OhdG: 8-hydroxy-2' -deoxyguanosine; ns: non significant. All values are presented as mean ± SEM. Statistical analysis by one-way ANOVA followed by Dunnett’s multiple comparison. ^a^*p* < .05, ^b^*p* < .01 and ^c^*p* < .001.

### 3.10. Effect of crocetin on NF-κB p50, NF-κB p65, and PKC activity

Crocetin dose-dependent treated group rats significantly (*p* < .001) abated the NF- κB p65 and NF-κB p50 activity (Figure 3). CT-PLGA-NPs exhibited the down-regulation of NF-κB p65 (142.3 ± 2.09) and NF-κB p50 activity (122 ± 3.82).

A similar momentum was observed in the PKC activity. PKC activity was alleviated via Crocetin intake as compared to the DM control group rats (Supplementary Figure 6).

### 3.11. Effect of crocetin on renal fibrotic factor

**Figure 5.**
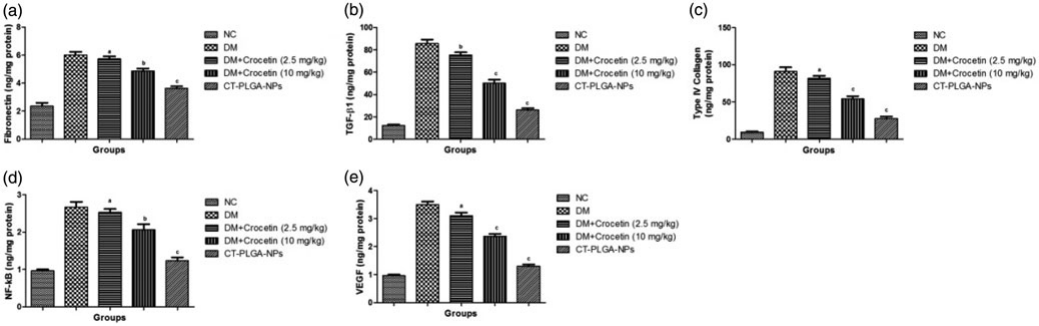
The effect of crocetin on fibrotic and inflammatory parameters of diabetic and non-diabetic rats. (a) fibronectin, (b) TGF-β1, (c) Type IV collagen, (d) NF-kB and (e) VEGF method as described in material and methods. TGF-β1: Transforming growth factor beta 1; NF-kB: Nuclear factor kappa; VEGF: Vascular endothelial growth factor; ns: non significant. All values are presented as mean ± SEM. Statistical analysis by one-way ANOVA followed by Dunnett’s multiple comparison. ^a^*p* < .05, ^b^*p* < .01 and ^c^*p* < .001.

Figure 5 illustrated the effect of crocetin on renal fibrotic parameters. DM group rats showed the increased level of fibrotic parameters such as fibronectin (6 ± 0.42), TGF-β1(85.86 ± 3.84) and Type IV collagen (91.04 ± 4.32) and CT-PLGA-NPs significantly (*p* < .001) reduced the level of fibronectin (3.2 ± 0.21), TGF-β1 (26.43 ± 2.04) and Type IV collagen (27 ± 1.04) at the end of the experimental study.

**Figure 6.**
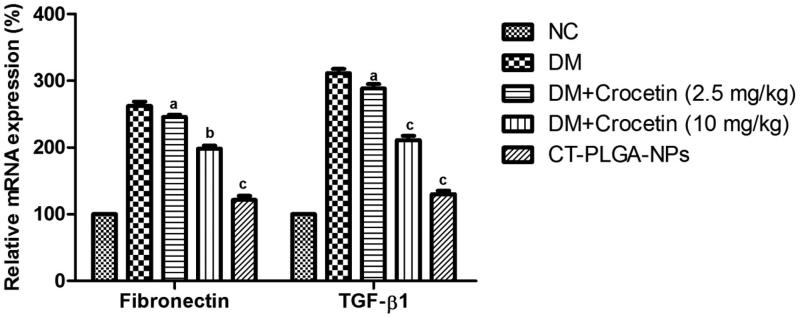
The effect of crocetin on relative expression of fibrotic parameters of diabetic and non-diabetic rats. Method as described in material and methods. TGF-β1: Transforming growth factor beta 1; NF-kB: Nuclear factor kappa; VEGF: Vascular endothelial growth factor; ns: non significant. All values are presented as mean ± SEM. Statistical analysis by one-way ANOVA followed by Dunnett’s multiple comparison. ^a^*p* < .05, ^b^*p* < .01 and ^c^*p* < .001.

Inflammatory parameters such as fibronectin and TGF-β both were boosted in the DM group rats and dose-dependent treatment of crocetin significantly (*p* < .001) down-regulated the level of both inflammatory parameters (Figure 6).

### 3.11. Effect of crocetin on antioxidant enzymes

Supplementary Figure 4 demonstrated the effect of the crocetin on the level of the antioxidant enzyme. During DM, the level of an endogenous antioxidant marker such as MDA and GSH was altered and the same level was observed in the DM control group rats. The MDA level was decreased and GSH level was increased and dose-dependent treatment of crocetin significantly (*p* < .001) altered at the end of the experimental study.

A similar effect was observed in the nitrotyrosine and 8-OhdG level, the level of both parameters were increased in the DM group rats and dose-dependent treatment of crocetin significantly (*p* < .001) decreased at end of the experimental study.

### 3.12. Effect of crocetin on histopathology

The normal control group rats showed the normal agriculture of renal tissue. STZ induced group rats showed the development of inflammatory necrosis cells, increase the size of Bowman capsules and deposition of fat droplets and CT-PLGA-NPs group rats showed the reduced size of Bowman capsules and fewer necrosis cells.

## 4. Discussion

Crocetin is a natural apocarotenoid dicarboxylic acid (present in *Gardenia jasminoides* and crocus flower), having a bitter taste (Moraga et al., 2004). This is the first investigation to identify the preventive effect of crocetin against renal injury in diabetic rats. The antidiabetic potential of crocetin might include increasing insulin secretion. Moreover, we observed that crocetin treatment down-regulated the fibrotic stress and renal inflammation and maintained the renal functions in DM rats. On the basis of the result, we can conclude that crocetin is a beneficial effect against diabetic nephropathy. The current experimental study suggests that crocetin may exert these beneficial effects by down-regulating the TGF-β1 and NF-κB/MCP-1 signaling pathways.

Various researchers suggest that the STZ-induced diabetic rats are the best model for the DM and induce diabetes similar to the human (Dâmaso et al., 2015). Several researchers suggest that the insulin secretory deficiency and destruction of the β cell are the main pathological features of STZ-induced diabetic rats (Tschöp and Heiman 2001; Bugger and Abel 2009). These show similar symptoms of diabetes like human viz., body weight, hyperglycemia, polyuria and polydipsia, andare the precursor of renal injury. In the current experimental study, we observed the increased blood glucose level, SCr, BUN, albuminuria, urine volume and reduced the body weight, plasma insulin in diabetic control group rats. In view of that, fibrosis, structural abnormalities and renal inflammation were higher in this group of rats. Especially, to reduce the intrinsic nephrotoxicity effects, we acquired the data at a regular interval after the STZ treatment when the kidneys have recovered from the acute renal injury. Consequently, we can say that renal injury in our experimental model was solely due to diabetes.

Previous researches suggest that the pro-inflammatory cytokines such as IL-6, TNF-α, and IL-1β are considered as the central mediators for ruling the inflammatory biomarkers and overproduction of these cytokines support the expansion of diabetes linked inflammation (Oliveira et al., 2008), coagulation (Silva et al., 2017) and endothelial dysfunction (López-Bojórquez et al., 2004). During DM, the overproduction of TNF-α decreased the insulin sensitivity by affecting the insulin receptor (Wachlin et al., 2003). It also induced the activation and accumulation of neutrophil, which further boosts the immune disorder in diabetic individuals (Cavelti-Weder et al., 2011). IL-6 demonstrates the effect on glucose metabolism by altering the IRS, Glut-4, and insulin receptor (Mark et al., 2004), which support in boosting the rate of insulin resistance (Kahles et al., 2014). In the current experimental study, we observed that crocetin treatment significantly (*p* < .001) reduced the production of IL-6, TNF-α, and IL-1β (mRNA expression) in renal tissue (Rotter et al., 2003). On the basis of the result, we can conclude that crocetin attenuated the renal inflammatory reaction via reducing the inflammatory cytokines.

Monocytes/macrophages, both activated, start the secretion of reactive oxygen species, nitric oxide, platelet-derived growth factor, lysosomal enzymes, interleukin, TGF-β and tumor necrosis factor-α, are promoting the renal injury (Abo-ouf et al., 2013). Platelet-derived growth factor (PDGF) excites fibroblast production and IL-1 induces the expression of TGF-β (profibrotic cytokine) in fibroblasts (Sun et al., 2017). In the diabetes mellitus, local growth factor viz., TGF- β are playing a significant role in the pathogenesis of nephropathy. TGF-β1 is a significant regulator of ECM due to induction of expression of fibronectin and type IV collagen in mesangial cells and further induced the renal dysfunction including albuminuria (Sun et al., 2017). Our result clearly illustrated that crocetin significantly reduced the expression and production of TGF-β1, which afterward reduced the fibronectin and type IV collagen and, therefore, improved albuminuria. On the basis of the result, we can conclude that crocetin could be restructured fibrosis occurred during diabetic nephropathy. Several studies suggest that the inflammatory mechanism may play an important role in the expansion of diabetic nephropathy based on the pathological findings of inflammatory cell infiltration in diabetic renal tissues. Inflammatory cells such as macrophages or monocytes are commonly present in the diabetic kidney (Reddy et al., 2014). They are secreted into the bloodstream and pull towards the target tissue via a process arbitrated through numerous chemokines such as MCP-1 (Piemonti et al., 2002). In the renal tissue, MCP-1 is present in tubular epithelial and mesangial cells and also involved in the pathogenesis of various renal diseases such as diabetic nephropathy (Giunti et al., 2010). The level of MCP-1 and microalbuminuria significantly increased in diabetes (type I). These findings suggest that the MCP-1 may play a significant role in the pathogenesis of diabetic nephropathy by inducing the inflammatory cell infiltration (Buraczynska et al., 2010). In the current experimental study, MCP-1 was considerably increased and supported the renal inflammation to be up-regulated, which resulted in the rats being at high risk of renal injury and its complications. Meanwhile, crocetin treatment clearly showed the reduced level of renal MCP-1, which suggests that crocetin having a beneficial effect against inflammation by reducing the macrophages and monocytes activation and down-regulating the recruitment of monocytes.

During diabetic nephropathy, the level of the BUN, creatinine and urine output considerably increased and crocetin treatment significantly (*p* < .001) reduced the BUN, creatinine and urine output and suggest the benefitting effect on renal functions. Crocetin 2.5 and 5 mg/kg b.w. treatment exhibited the anti-inflammatory effect but crocetin 10 mg/kg b.w. treatment showed the anti-inflammatory effect along with anti-fibrotic effect. The result suggests that the higher dose of crocetin treatment attenuate renal fibrosis for diabetic individuals.

Several factors such as oxidative stress, hyperglycemia, and growth factors activated via NF-κB and the concentration of NF-κB boosted during the human diabetic nephropathy condition (Baker et al., 2011). Several researchers suggest that the activation of NF-κB is consequently regulating the inflammation mediator viz., IL-6 (Serasanambati and Chilakapati 2016) and TNF-α (Ramakrishnan et al., 2013). In the current experimental study, crocetin significantly (*p* < .001) down-regulated the protein production and mRNA expression NF-κB p50 and p65, which in turn reduced the inflammatory cytokines formation and restructured renal inflammatory injury. It is suggested that the nuclear level of NF-κB p65 is positively related with renal activation of NF- κB pathway in diabetic rats. The possible mechanism of action may be due to its anti-inflammatory nature by down-regulating the NF-κB expression.

On the contrary, NF-κB is the significant transcription regulator for fibrotic factors such as fibronectin and TGF-β1 (Malik et al., 2017). Consequently, crocetin treatment considerably reduced the activation of NF-κB also down-regulating the fibronectin and TGF-β1 expression, which subsequently improved the renal fibrotic stress. In the current experimental study, we also estimated the PKC activity, the DM group rats showed the increased concentration of PKC and crocetin treatment significantly (*p* < .001) inhibited the PKC activity at dose-dependent manner. The reduction of PKC activity induces the diminution in the activation of NF-κB, which in turn limited the transcription of its downstream factors (Aveleira et al., 2010). Our data clearly revealed that crocetin treatment reduced the renal PKC activity. Consequently, it may be the intake of crocetin that reduced the renal PKC activity and abated the activity and expression of NF-κB, which further considerably down-regulated the production of fibrotic and inflammatory factors and finally improved the renal functions.

## Conclusion

We can conclude that crocetin provided the renal anti-fibrotic and anti-inflammatory effect in diabetic rats. In the current experimental study, we have found that crocetin down-regulated the production and expression of fibrotic factors viz., TGF-β1 and fibronectin and inflammatory cytokines including MCP-1 and TNF-α in renal. Crocetin also considerably abated NF-κB expression activation and PKC activity. On the basis of the experimental study, we can say that crocetin rich food and diet might be obliging for the prevention or improvement of diabetic nephropathy. Further molecular studies are necessary to scrutinize its safety before it is used for human.

## Supplementary Material

Supplemental Material
