# Supplementary material for: Design and optimization of crocetin loaded PLGA nanoparticles against diabetic nephropathy via suppression of inflammatory biomarkers: a formulation approach to preclinical study
Source: Drug Deliv. 2019 Sep 14;26(1):849–59. doi: 10.1080/10717544.2019.1642417 (PMC6761602; doi:10.1080/10717544.2019.1642417)
Supplement: Supplemental Material [file IDRD_A_1642417_SM9718.docx]

**
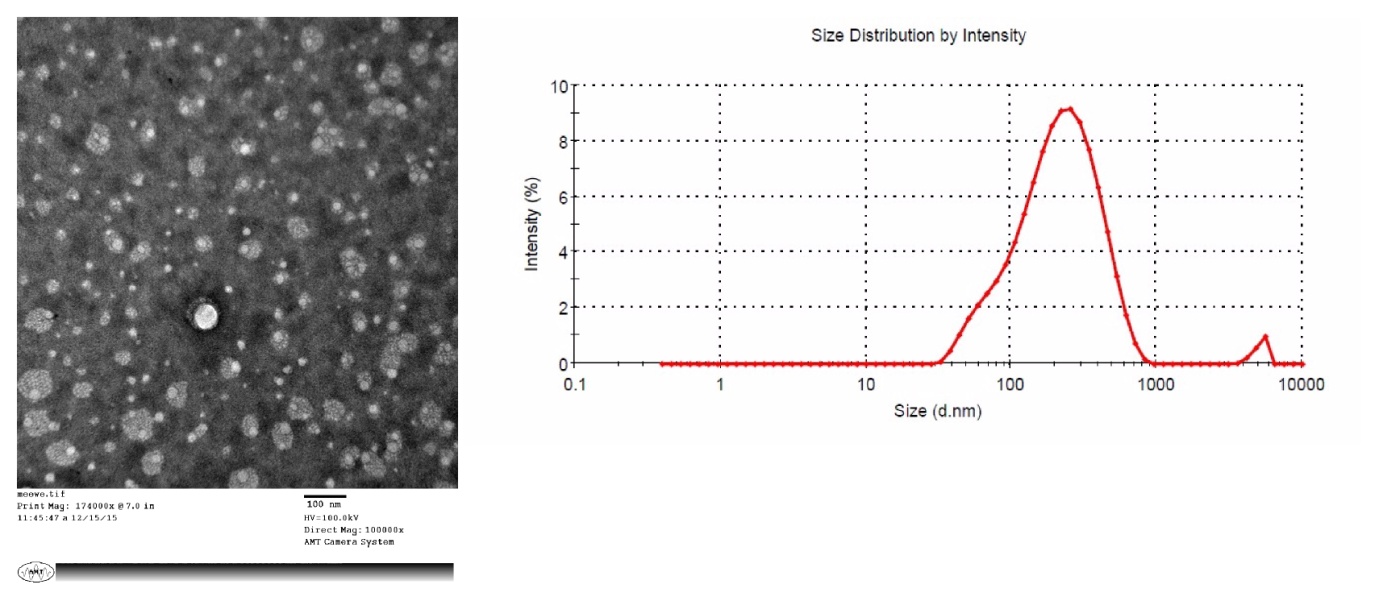
Supplementary figure 1:** characterization of CT-PLGA-NPs. (a) transmission electron microscopy (b) particle size distribution of prepared PLGA loaded crocetin nanoparticles.

**
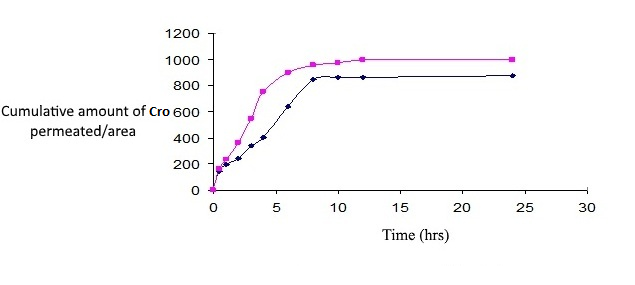
Supplementary figure 2:** showed the drug release pattern of crocetin and nano-formulation of crocetin.

**
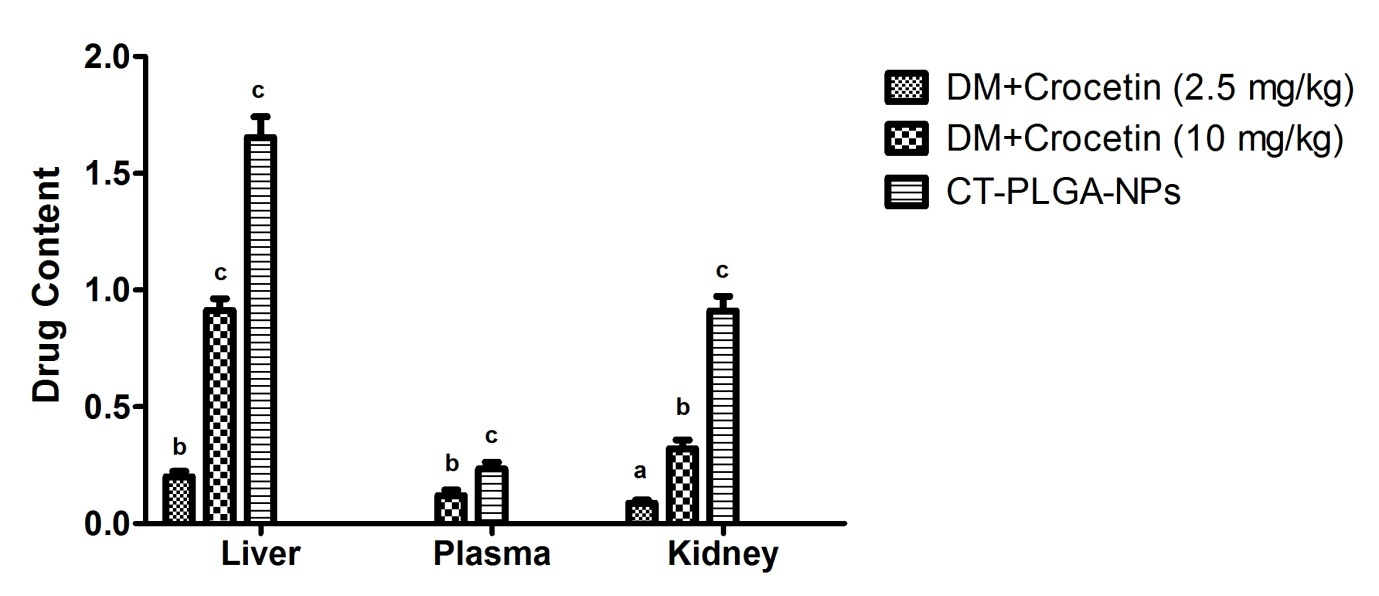
**

**Supplementary figure 3:** Crocetin content in Hepatic, Plasma and Renal from diabetic and non-diabetic rats. non-diabetic and diabetic rats did not show the content of crocetin. Data are the mean ± SD. ^a^P < 0.05. ^b^ P< 0.01 and ^c^P < 0.001.

**
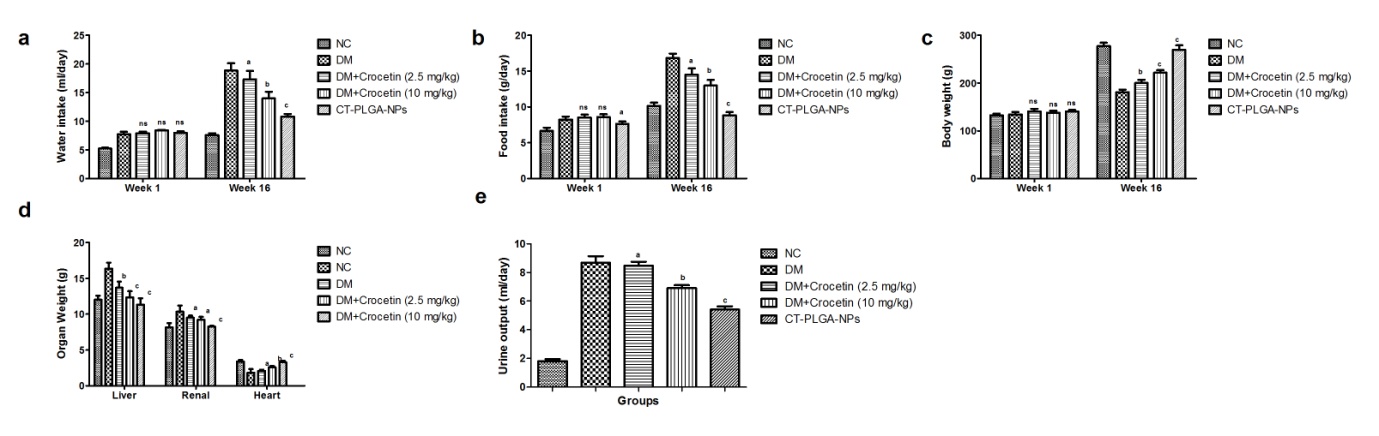
**S**upplementary figure 4:** showed the effect of crocetin and CT-PLGA-NPs on feed intake, water intake, body weight, organ weight and urine output of diabetic and non-diabetic rats. a: water intake, b: food intake, c: body weight, d: organ weight and e:urine output method as described in material and methods. ns=non significant. All values are presented as mean ± SEM. Statistical analysis by one-way ANOVA followed by Dunnett’s multiple comparison. ^a^p < 0.05, ^b^p < 0.01 and ^c^p < 0.001.

**
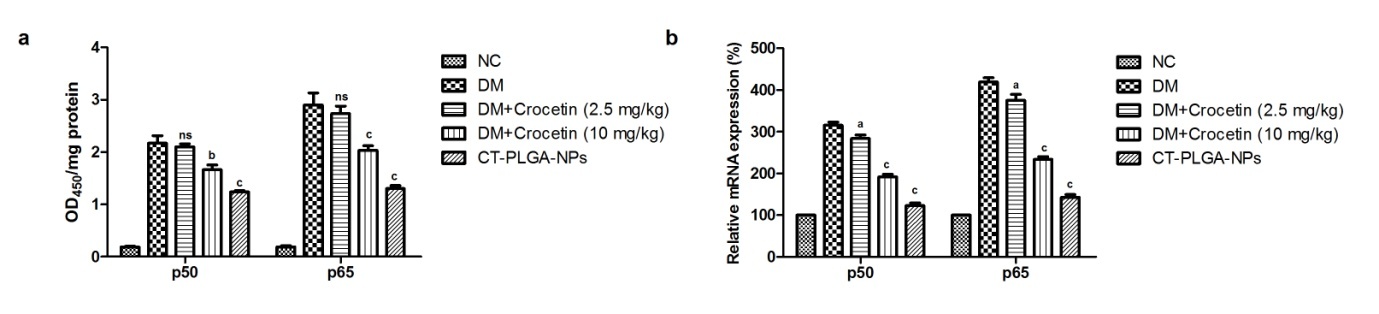
Supplementary figure 5:** showed the effect of crocetin on relative expression of NF-Kb p65 and p50 of diabetic and non-diabetic rats. Method as described in material and methods. ns=non significant. All values are presented as mean ± SEM. Statistical analysis by one-way ANOVA followed by Dunnett’s multiple comparison. ^a^p < 0.05, ^b^p < 0.01 and ^c^p < 0.001.

**
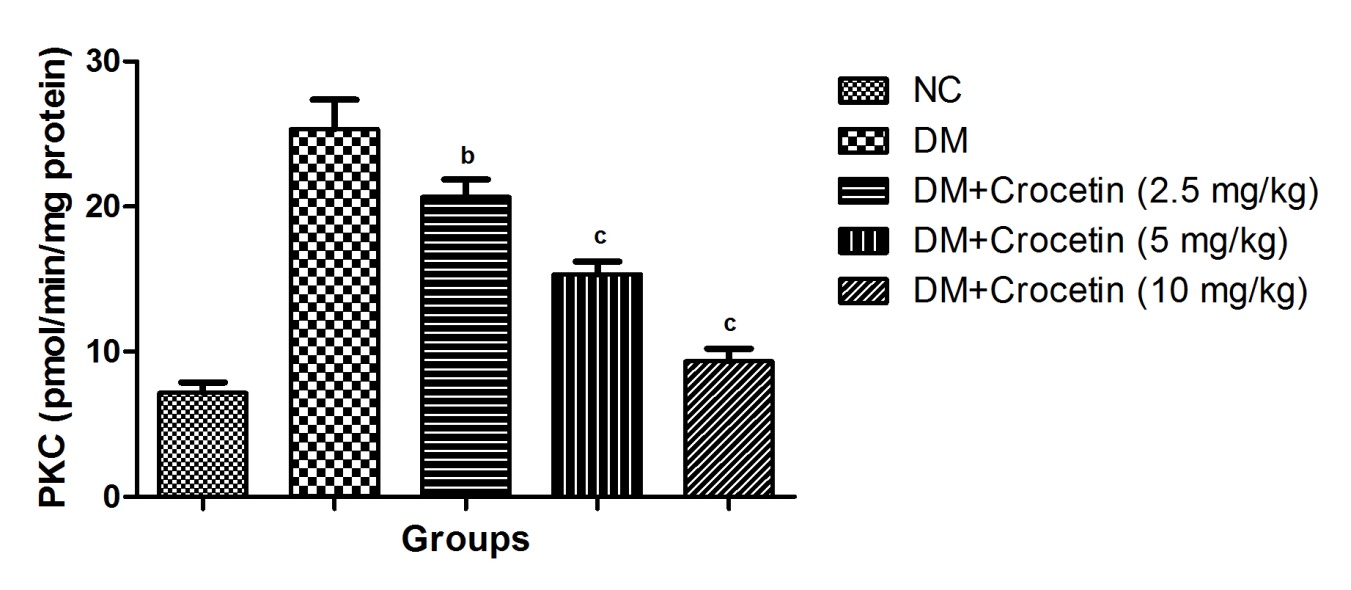
Supplementary Figure 6:** showed the effect of crocetin on PKC activity of diabetic and non-diabetic rats. Method as described in material and methods. PKC=, ns=non significant. All values are presented as mean ± SEM. Statistical analysis by one-way ANOVA followed by Dunnett’s multiple comparison. ^a^p < 0.05, ^b^p < 0.01 and ^c^p < 0.001.


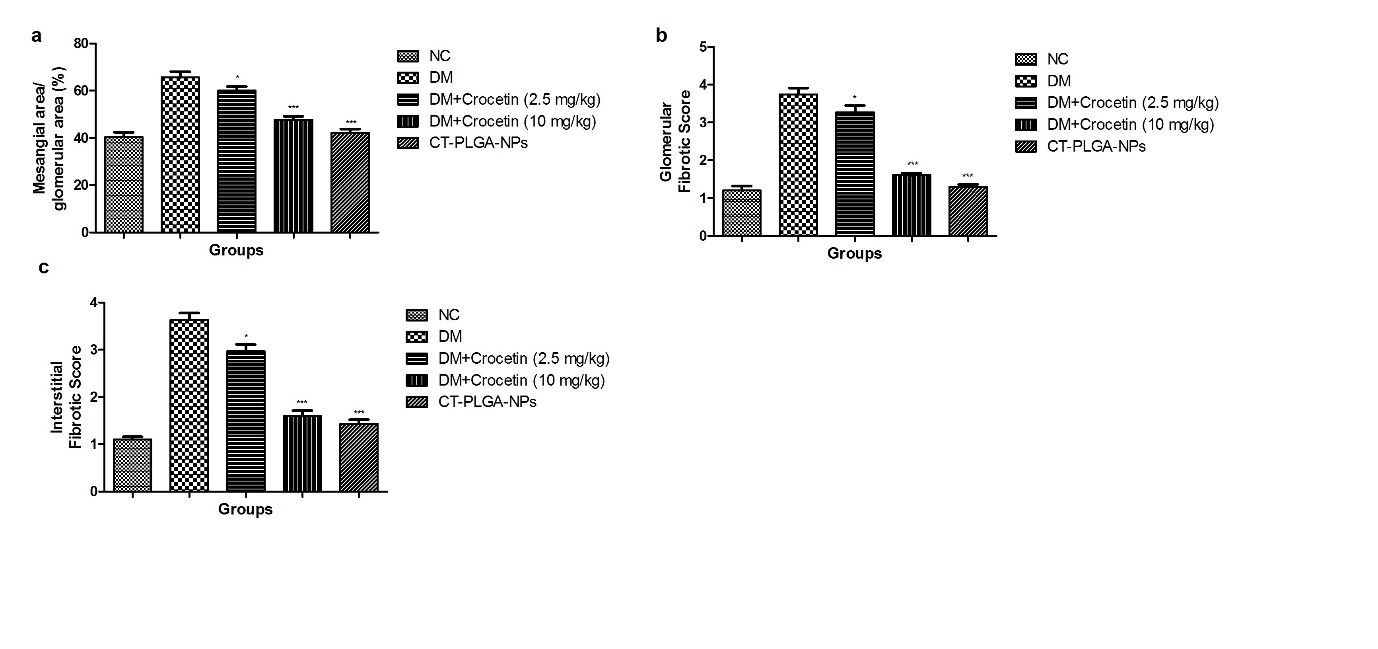


**Supplementary figure 7:** showed the renal histopathology parameter after treatment with or without crocetin.
